# Abstracts and Gleanings

**Published:** 1880-10-20

**Authors:** 


### ABSTRACTS AND GLEANINGS.

**Successful Removal of a Uterine Fibroid with Thomas' Scoop or Spoon.**—The following case was reported in the *Ohio Medical Record* for April, 1880, by E. W. Howard, M.D., of Akron, Ohio:

Mrs. B., aged fifty-two years, the mother of three children, the youngest twenty-six years of age, had for four or five years been the subject of frequent and severe uterine hemorrhages, so that at times her life had been almost despaired of. During this time, she had been under the treatment of a gynecologist, who attributed her flooding to change of life.

In May last, being reduced to the last extremity, she was placed in other hands. Her attendants suspected a uterine fibroid, and upon dilating the mouth of the uterus with sponge-tents found one of considerable size, of the interstitial and sub-mucous variety, very firmly attached to nearly the whole of the fundus and left side of the uterus. Her physicians decided to remove it with the ecraseur, and anæsthetized her with ether, believing that in her exsanguine condition, with evident heart trouble, the use of chloroform might prove fatal. But she did not get beyond the stage of excitement, and, as they said, fought like a tiger the whole time, a period of nearly three hours. The ecraseur was applied, but when tightened up the chain broke. A silver wire was tried, with the same result. No effect had been produced upon the tumor, and further proceedings were suspended. Her condition was then alarming, a heart-clot being feared.

When I saw her, I informed her that any further effort, in her condition, would prove fatal, and advised controlling the hemorrhage with ergot, and trying to build her up with iron, quinine, and other tonics, and liberal diet, trusting to the turn of life to give more permanent relief. She improved very slowly, and after several weeks was removed to Akron and placed in my charge. The same treatment was continued, but the hemorrhages were renewed. Ergot failed to arrest them, and I gave dilute sulphuric acid, which did better. I then gave large doses of ergot, with the double purpose of partially extruding the tumor from the uterus, and also arresting its growth. It was only partially extruded, but no effect was produced upon its growth. The hemorrhages became more alarming. I could not make the slightest and most careful digital examination without provoking flooding. In this state of affairs, I decided to operate at once, but with the gravest apprehensions as to the result, especially fearing the anæsthetic and hemorrhage. Accordingly, on October 28th, 1879, assisted by my son, Dr. H. C. Howard, and my friends, Drs. Thomas and L. S. Ebright, I proceeded to remove the tumor in the following manner: After placing her upon the table, we commenced giving her ether, and soon she became almost unmanageable, and fought as before. Then we gave her the A. C. E. mixture—alcohol, one part; chloroform, two parts; ether, three parts—and in one minute she became perfectly quiet. She was then placed in Sims' position, Sims' speculum used, the tumor grasped with strong vulsellum forceps, and Thomas' spoon was gently insinuated between the walls of the uterus and the tumor, when, with a gentle pendulum motion, it was rapidly removed without the loss of an ounce of blood. It weighed eight ounces. The effect of the chloroform passed off without a single unfavorable symptom, and she was removed from the table and placed comfortably in bed in just sixteen minutes from the time she first went upon the table. She has made a rapid and most satisfactory recovery without an untoward symptom.—*Med. and Surg. Rep.*

**Diseases of the Eye Occurring in Connection with Pregnancy.**—Mr. Henry Power contributes an important series of articles on diseases of the eye in connection with pregnancy, to the *Lancet*, May 8th, 15th, 29th, 1880. He commences by reviewing the physiological changes induced by pregnancy, and concludes that the quantity of blood, though increased absolutely, is not relatively, and that in pregnancy a condition of general anæmia is far more commonly met with than one of hyperæmia. From an examination of the various cases which have fallen under his notice, he would classify the diseases of the eye in connection with pregnancy under three heads, namely: 1. Affections depending on general anæmia and exhaustion; 2. Those consequent on some special lesion of the nervous system; 3. Those depending upon, or rather associated with, albuminuria. Among diseases attributable to exhaustion, the most common are ulcers of the cornea, which may be either spontaneous or arise from some slight injury. They are often central, are slow in their progress, and are not usually dangerous. The treatment may be summed up in two words, rest and tonics. The former indication may be fulfilled by a two or four-grain solution of atropine or eserine, and the application of a pad of cotton-wool and a bandage; the latter by quinine. During lactation, a more dangerous form of ulcer is often met with, causing destruction of the cornea, with eventually atrophy of the globe. Paracentesis corneæ is often required in such cases. Another sign of exhaustion in pregnancy is impairment of the power of accommodation, due to enfeebled action of the ciliary muscle. Glasses, in suitable cases, will necessarily be required, but much good also may be effected by tonics, especially strychnia in small doses. As regards special lesions, the author has witnessed what he considers an increased tendency to lachrymal abscess, and to the development of cataract. Lesions of the nervous system, or lesions implicating the nervous apparatus of the eye generally, he divides into two groups, the intra- and the extra-ocular. The former affect the retina, the latter the optic nerves, chiasma, optic tract, and central ganglia. The retinal affections are almost limited to cases of abuminuria, though also cases of hemorrhagic glaucoma, and miliary hemorrhages unconnected with albuminuria in pregnancy have been noted by Galezowski. Two cases are recorded by the author, in which retinal hemorrhages during pregnancy passed off harmlessly, and one in which they were of fatal significance. As regards the treatment of such cases, it is the same as that of retinal disease generally, no special treatment being demanded for the eyes. Some writers have recommended the induction of premature labor in these cases, but the author considers more data are required before a positive opinion can be pronounced, more especially as to the period when labor could best be induced. As regards intracranial diseases in pregnancy, the author suggests they could almost be classed under the head of "anomalous affections." He gives the histories of cases of partial or complete loss of vision from post-partum hemorrhage, and explains such either by abolition of the circulation in some portion of the cerebrum, or by some lesion of the delicate tissue of the central nervous system from sudden diminution of pressure. Many such cases eventually resolve themselves into atrophy of the optic disc. A comparison between this condition and the occurrence of chorea in pregnancy may perhaps be instituted.—*Philadelphia Med. and Surg. Rep.*

**Abortions.**—We extract from Dr. Stevens' paper before Cincinnati Medical Society the following:

A capital book by Prof. A. R. Simpson has recently been issued by Messrs. Black, from Edinburg. In the management of cases of abortion, says Prof. S., where "the stage of expectancy is clearly over," and the conditions otherwise call for active interference, he recognizes simply two main indications to be fulfilled, viz.: 1st, to restrain hemorrhage, and, 2d, to complete the evacuation of the uterus. For the first indication Prof. Simpson only proposes ergot and the plugging of the genital canal. He either had not heard of the method of controlling uterine hemorrhage by hot water injections, or has not come to accept them as reliable.

In regard to the use of ergot in abortions, my own experience has been that this drug, seeming to expand itself upon the cervical portion of the uterus, causes such a firm and painful contraction of that portion of the organ as to materially interfere with the ultimate removal of retained fragments of placenta or membranes. I have to say, however, further that with quite an experience with abortions, I have scarcely ever had a seriously troublesome hemorrhage.

To meet the second indication. Dr. Simpson directs the following order of procedure:

1. To anæsthetise the patient as a general rule.

2. To push down the uterus from above by pressure with the left hand.

3. To pass into the uterine cavity one or more fingers to sweep the cavity, peel off any adherent mass, and force the whole through the cervical canal. The use of all such instruments as curettes, wire loops, forceps, etc., are deprecated. Except when all adhesions have been separated by the finger the removal of the loosened body may be sometimes facilitated by some form of forceps.

Should these measures prove insufficient, then, as a further and final resource, Prof. S. says:

Seize the anterior lips of the uterus with a volsella and thereby drag down the cervix from below so as to place the uterus under the certain and absolute control of the operator. I take it the use of the volsella after this method is about all in the directions of Dr. Simpson, clever as his chapter is, that is not amongst the usual procedures of the experienced obstetrician.—*Obstetric Gazette.*

**Acidity as a Caus. of Sterility.**—In many cases of sterility the cause is not discoverable. The conditions for impregnation are apparently all present—the anatomical configuration of the female genitalia is perfect, and the male fluid teems with lively spermatooza—but the most honest effort is unattended with the desired result. In a recent paper read by Dr. Charrier before the Paris Societe de Medecine, and published in the *Bulletin de Therapeutique*, the possible hindrance in such cases is pointed out. The paper closes with the following conclusions :

1. In some rare cases, in women who are otherwise quite well, the utero-vaginal secretions are quite sour, as is seen by their reddening litmus.

2. This acid may prove an absolute obstacle to fertility, as spermatooza are killed in even a slightly acid medium.

3. This abnormal state is to be remedied by an alkaline treatment, by means of alkaline drinks and baths, and tepid alkaline injections.

4. When this acid condition has been neutralized, conception may take place. (Two cases in point are detailed.)

5. This disappearance of acidity under the influence of alkaline treatment may explain the success which is obtained at alkaline and sulphuro-alkaline mineral water establishments in the treatment of sterility.

In a note in the *Bulletin* of June 30th, Prof. Pajot entirely confirms this statement, and says that for many years past he has been in the habit of prescribing injections of Vichy water in these cases of acid vaginal discharges. He observes that in fair women, and especially those with a red complexion, and more rarely in brunettes, the acidity of the secretions sometimes reaches such a point that, in spite of the extremest cleanliness, the acid odor is perceived during the passage of the speculum. Dr. Charrier says that the best liquid for injection in these cases is that devised by Byasson (water 1000 grammes, the white of one egg, and fifty-nine grammes of phosphate of soda), in which he was able to keep spermatozoa alive for twelve days at a temperature of 36° C.—*Mich. Med. News.*

**Influence of Tobacco Smoking upon Health.**—In the *Revue d' Hygiene* for November 15, 1879, Dr. Decaisne observes that the excessive use of tobacco causes in some subjects intermission of the beats of the heart and radial artery. Out of eighty-eight smokers who came under his observation during a period of three years, he found twenty-one cases of intermittent pulse without any organic lesion of the heart. He states—

1. That none of the subjects who came under his observation had organic disease of the heart.

2. That none of them were in a state of health favorable to the development of intermittence in the beats of the heart.

3. That in nine cases the complete abstention from smoking tobacco was sufficient to restore the cardiac rhythm and the system to their normal condition.

He therefore says that the abuse of tobacco produces in some subjects a condition which he terms *nicotism* of the heart, which is manifested by intermission in the beats of that organ, and in the pulsation of the radial artery. It is sufficient in some cases to suppress or diminish the use of tobacco in order to stop or diminish the irregularity in the heart's action.

Dr. Delaunay, in commenting upon Dr. Decaisne's paper in the *Revue d' Hygiene* for January 15, 1880, calls attention to the influence of the emanations from tobacco upon pregnancy. In a poor district of Paris, there is a manufactory of tobacco in which two thousand women are employed. According to the evidence of a midwife, who has attended a great number of women employed in this factory during their confinements, they are peculiarly liable to miscarriages, which they attribute to the influence of the exhalations from the tobacco. Some of the women, who are not altogether dependent upon their earnings from the factory, leave it when they become pregnant until after their confinement. One woman, who had twice miscarried while working at the factory, left it when she was in the fifth month of her third pregnancy. Her child was born alive at the proper time, but died soon after its birth. The mother did not return to the tobacco factory, and her fourth pregnancy terminated in the birth of a healthy child, who survived. During her first three pregnancies, she suffered much from obstinate vomiting—due, perhaps, to the action of the tobacco.

From these and similar data, Dr. Delaunay deduces the following conclusions:

1. Tobacco has a pernicious influence upon the health of children and mothers.

2. It impairs the health of pregnant women and causes miscarriage.

3. It has the same noxious effect upon children weak from their birth.

4. It diminishes the quantity of milk and alters its quality for the worse, and, consequently, prevents the proper growth of the child, who, indeed, often dies a victim to its mother's occupation.—*Med. and Surg. Rep.*

**Obstetric Practices Among the Indians.**—Dr. G. A. Moses, in the St. Louis *Courier of Medicine*, gives the following account of obstetric practices among the Indians: An Indian woman of the Kiowa tribe—one of the wildest tribes, which has come scarcely at all in contact with the whites—had been in labor for three days, and it being apparent to the friends and midwife squaw that successful natural delivery was impossible, and that under the native treatment by incantations, beating of tom-toms, etc., the woman's strength was becoming rapidly exhausted, assistance of the post medical officer was desired. It was only after several visits to the wigwam that the doctor finally was allowed to make a very hasty and imperfect digital touch. The head was arrested in the cavity. After still further delay, he was permitted to apply the forceps, which, to the intense amazement of the lookers-on, drew forth a living infant. As soon as this was effected, the physician was rudely pushed aside, and the Indian midwife took charge of her case, compelling the woman to rise to her feet. She was sustained in a bent posture, grasping with both hands the centerpole of the tent; then the squaw proceeded to carry out methodically Crede's method of expresssng the placenta by compressing the uterus through the abdominal walls, with both hands pressing in the direction of the pelvic cavity, until the placenta appeared at the vulva, when it was seized with one hand and withdrawn; the patient was then allowed to resume recumbency, and a highly ornamented buckskin bandage was adapted to pelvis and abdomen; this was drawn snugly by buckles and straps. The doctor says it looked as though it had been in use some time, and was a most perfect-fitting bandage. The patient made a good recovery, and the white man's "iron hooks" are established in reputation among the band.

If the Indian mother gives birth to twins, only one is allowed to live. In case of the birth being male and female, the latter is given to an old squaw, and nothing further is heard of the luckless papoose. In case of both being of the same sex, the feeblest is put out of the way.—*Boston Medical Journal.*

**Cupping in Carbuncle.**—Dr. Hunt writes to the Chicago *Medical Journal:* In the early period of my practice, some forty years ago, I used the cups in the treatment of local diseases more frequently than now. During this period, I had to treat a bad case of carbuncle, situated on the back of the neck of an old man. While dressing it one day, it struck me forcibly that cupping would be just the treatment for this case. Calling for a large goblet and some cotton, I applied it as a cup, after expanding the air by burning cotton in it. The effects were truly wonderful, drawing out from the interior of the tumor a large amount of pus and corruption, which gave immediate relief. The night following, the old gentleman rested for the first time. Since this experiment—the first one of which I ever heard or knew—I have relied mainly on the cups for the local treatment of carbuncle. It fulfills the most important indications in the local treatment of this often troublesome and sometimes dangerous disease. It relieves tension and pain, and limits gangrene of the cellular tissue. It materially shortens the time of cure. With appropriate general treatment, the disease is shorn of half its pain, duration and danger. The cups may be applied once or twice a day, or even oftener. If resorted to in the early stage, the scalpel or lancet should be used to induce a free flow of blood. Mere dry cupping at this time would increase the flow of blood to the tumor without relief. I would caution against too severe cupping until pus is formed. I more often use a large, blunt-rimmed tumbler or goblet than any other kind of cup. The size of the opening of the cup should be, if possible, sufficiently large to cover the base of the tumor. An air-pump attached to the cup, if at hand, would be much more manageable and convenient; but the tumbler and cotton may be used with almost equally good effect, if adroitly done, besides having this advantage—of being always available.—*Chicago Medical Times.*

**Uterine Dyskinesia and the Treatment of Displacements.**—Uterine dyskinesia is a new gynecological term introduced by Dr. Graily Hewitt, and used to express the difficulty in walking that accompanies certain uterine diseases. In a report upon sixty-seven cases of uterine distortion or displacement coming under Dr. Hewitt's care, he noticed this symptom as occurring with remarkable frequency. Physical exertion induces a temporary exaggeration of the difficulty; hence exercise is given up and helpless invalidism is likely to ensue. Another point noticed in these cases, which, by the way, were of persons of the better class, was the frequent existence of starvation. Not enough food was taken, and the uterine tissues softened and lost their tonicity. In many cases, nausea was also a frequent symptom of the uterine displacement. This nausea sometimes led to the taking an insufficient quantity of food; the result was starvation—the starvation in these cases being secondary to the uterine disease.

The treatment employed was largely hygienic. In some starvation cases, food was given every hour; sponge baths and friction to the skin were used. The postural method was largely followed. The patients were kept recumbent, in the dorsal position in the case of forward displacements; in the semi-prone position in cases of backward displacements. In cases of forward displacement, the cradle pessary; in backward, the Hodge pessary was employed. The sound was used at intervals to aid in restoring the uterus to proper shape when the organ was found hardened in its distorted shape. The treatment of the cases generally covered a long time, but eventually most of the patients were restored to health.—*N. Y. Med. Record.*

**Specular Examination of the Rectum.**—Sims' speculum, for examination of the rectum, is incomparably superior to all others. It is to be used in the same manner as for vaginal examinations, with the patient in Sims' lateral, or, accurately speaking, latero-prone position, and, when used in this way, it gives as good an exposition of the rectum as of the vagina. Its use is much facilitated by forcible dilatation of the anus by the method of Van Buren, but unless the dilatation is indicated as a part of the patient's treatment, this may usually be omitted. Applications to the rectum may be made through this speculum with as much ease as to the vagina or uterus. The operation for internal hemorrhoids also is much facilitated. When the operation to be performed is very far above the anus, the walls of the rectum may, by contact with one another, cause annoyance. This difficulty may be overcome by a device of Dr. A. J. Stone, of Saint Paul, Minnesota. Dr. Stone attaches to a soft rubber tube, armed with a stylet, a rubber bulb with very thin and flaccid walls, in such a manner that by blowing through the tube the bulb may be inflated. The speculum is first to be introduced. The collapsed bulb is then to be pushed by the stylet above the place of operation, inflated by blowing through the tube, and retained in place by slipping the tube, the stylet remaining in place back of the speculum, where it will be entirely out of the operator's way. All that part of the rectum below the inflated bulb will then be distended and accessible.—*Chicago Medical Gazette.*

**Treatment of Lung Diseases by Condensed Air.**—We reprint the following from the *Physician and Surgeon:* Dr. Pomerantsef read a paper on the above subject at the meeting of the Imperial Caucasian Medical Association. He has employed Waldenburg's apparatus in two cases, one of *pleuritis exudate* and the other of *emphysema pulm.* In the first case, the result was strikingly satisfactory: the exudate was absorbed, the cough ceased entirely; the stitches in the left side, where the exudation had taken place, disappeared; appetite improved, fever less, and the face of the patient, who had been anæmic and exhausted, assumed a healthy color. The circumference of the whole thorax increased six and a half centimeters, that of the affected side five centimeters; the capacity of the lungs increased four hundred and thirty cubic centimeters; the patient gained twenty-five pounds in weight. Time of treatment—two months and twelve days. In the second case, the treatment consisted in inspiration of condensed air, with expiration into the common atmosphere, and lasted three months. In this case, dyspnœa had been severe, but breathing soon became easy without abnormal effort of the thoracic and cervical muscles; the cyanosis and cough disappeared, and it was observed that the circumferential measurement of the thorax increased one centimeter, and inspiratory expansion also increased one centimeter, the capacity of the lungs two thousand one hundred and fifty cubic centimeters, and the patient gained six pounds in weight.—*Med. Obozpania.*

**Eczematous Crusts.**—Eczematous crusts in the nares are best removed with a solution of bicarbonate of soda, about twenty grains to the ounce of water, introduced in the form of spray, or in bad cases with the post-nasal syringe first and the spray after the larger crusts have been expelled. In some cases the crusts may have to be pulled out with forceps. If after removal of incrusted matter from the nares hemorrhage occurs from the exposed and excoriated membrane, a solution of carbolic acid and tannin may be used with great benefit. This is a good formula:

| | | |
|---|---|---|
| ℞ | Acidi carbolici C. P. .................... | ʒ ss |
| | Acidi tannici .................... | gr. xij |
| | Aquæ .................... | ℥ vj |
| M. ft. solutio. | | |

Sig. Use with the atomizer for the nose.

This should be used immediately after exposing the excoriated membrane, and within an hour a spray of chloride of sodium or bicarbonate of soda should be freely used, with the view of aiding nature to supply the required saline covering for the exposed superficial nerves.—*Med. Herald.*

**Metaphosphoric Acid a Delicate Test for Albumen in Urine.**—Dr. W. C. Grigg states, in the *British Medical Journal* for May 29, 1880, that he has recently made a series of experiments with nitric acid and metaphosphoric acid, and finds that the latter acid will demonstrate the presence of albumen after the former has ceased to give a reaction. His attention was first directed to it by Dr. Dupre, F.R.S. He believes it has long been known to chemists, and used by them as one of the most reliable tests, if not the most reliable, for albumen, but he is not aware that it has been used for clinical purposes.

In using it, care should be taken that the solution of metaphosphoric acid is freely made, and that no heat is applied to dissolve it, as it is a very unstable acid and readily decomposes. The plan he has adopted is to put a piece of metaphosphoric acid, about the size of a pea, into a drachm of distilled water. The urine can either be added to the solution or the acid solution to the urine. If there be a trace of albumen, the urine will immediately become turbid and of a milky-white color.—*Phil. Med. Reporter.*

**Administration of Iodide of Potassium.**—A most palatable way of administering the iodide to children, or to adults whose gastric membrane is easily irritated, is as follows in any dose required:

℞ Potass. iodidi........................................ gr. 10

Syrupi cydonii........................................ ℥j

M. Sig. In a glass of water.

I prepare the syrup of cydonium as follows:

℞ Cydonium (quince seeds)........................... 1 part
Cydonium malum (quince fruit)...................... 3 "
Simple syrup........................................ 4 "
Water sufficient.

Boil together until seeds and fruit can be crushed with a spoon, then boil until the mass becomes of the consistence of molasses. Strain through a fine sieve. The syrup prepared this way keeps as long as jelly. It is an elegant vehicle on account of its delicious flavor and its mucilaginous properties, giving us in one preparation a demulcent as well as a placebo.

I commend this vehicle not for the iodide alone but likewise for the nitrate of potassa and such drugs as are objectionable when given in a watery solution.—*Med. Herald.*

**Benzol in Whooping Cough.**—Dr. John Lowe writes to the *Lancet* (American edition for July) that for ten or twelve years he has used benzol as a remedy for pertussis, and that he has not found it to be of service until the acute stage has passed. After the first fortnight he gives the following: Benzol, 2 to 10 minims; tincture of hyoscyamus; compound tincture of chloroform to suit each case; mucilage of gum acacia, and water, in sufficient quantity. The benzol should be pure, although, barring the unpleasant odor, he has not found the less pure variety less efficacious.—*New Remedies.*

**Prolonged Absence from Food.**—Dr. J. C. Noyes, of Oshkosh, Wisconsin, reports, in the Boston *Medical and Surgical Journal*, a case of a man, aged 34 years, who has taken no food during the past forty-five days, or water, except to rinse his mouth, during the past nineteen days. "In fact," says Dr. Noyes, "has been unable to swallow either during those respective periods." Owing to his present condition, and the imperfect history obtained, a satisfactory diagnosis is impossible. An autopsy only would be conclusive. However, the facts above stated can be fully sustained.

Dr. Edwin Stewart, of Mendon, Michigan, reports, in the *Medical and Surgical Reporter*, a case of a woman, aged 66, who died on the 9th of July, having lived since her breakfast, on May 19th, fifty-two days, forty-eight of which were without food of any kind, except three crackers and five spoonsful of chicken broth.

A case is on record in this city where an individual lived fifty-six days without food. Since Dr. Tanner completed his fast, a number of such cases have been reported.—*Maryland Medical Journal.*

**Systemic Poisoning by the External Application of Carbolic Acid.**—The following case was reported in the Medical Times, by Dr. Comegis Paul, of this city :

A young convict, about 24 years old, complained of the excessive discomfort caused by a crop of herpes upon his right side, extending from the nipple to the axilla. The part was painted with a saturated solution of carbolic acid, with the effect of entirely relieving the pain. It was then dressed with vaseline. Two days after he asked to have the acid again applied. Within twenty minutes after it was done he became faint and dizzy, very weak in the legs, and exhibited all the signs of a general collapse. The condition lasted about half an hour when he gradually began to revive.

The surface covered by the carbolic acid was not more than five square inches, and the second application came in contact with only a partially denuded cuticle of much smaller extent.—*Med. and Surg. Rep.*

**Anthrax of Fruit-Trees.**—Dr. Burrill (in American Association of Science) stated that the wide-spread disease of the pear-tree, known as fire-blight, and that of the apple-tree, known as the twig-blight, are due to a common cause. This is a living organism which produces butyric fermentation of the material stored in the cells, especially those of the liber. The organism is similar to, if not identical with, the butyric vibrione of Pasteur, and the *bacillus amylobacter* of Van Tieghem. It assumes various shapes during development, but its characteristic form is that of two oblong joints with rounded ends, the proportions of each joint being .002 mm. by .003 mm. They are shorter and thicker than the bacterium termo, and move more slowly. A large number of experiments resulted in showing pretty conclusively that these organisms conveyed the disease from tree to tree. The most conspicuous change in the tissues of the affected plant, revealed by the microscope, is the almost total disappearance of starch from the cells.

**Method of Restoring the Asphyxiated.**—When the body is elevated to an angle of forty-five degrees or more, with the head down, the abdominal viscera fall against the diaphragm and force the air, mucus and all foreign matter from the lungs and air-passages; the blood also flows to the brain, right side of heart and lungs, stimulating those organs. Reversing this movement, the abdominal organs fall away from the diaphragm, drawing it along with them. The air rushes into the lungs to fill the vacuum created. The blood flows to the right and left side of the heart and lower extremities, in this way imitating the normal movements of respiration and circulation as nearly as may be.

The details as to how to execute the movements will suggest themselves to almost any one of ordinary sense. Children can be caught up in the arms of a person and the movements executed.—DR. SHEPPARD, *in Detroit Lancet.*

**Podophyllin.**—All who have been accustomed to prescribe podophyllin in pills will agree as to the impossibility of preventing occasional disastrous effects. But this is the fault of the form of administration—not of the drug. From a very long and extensive experience, I can confidently affirm that none of the accidents and inconveniences which so commonly attend the administration of podophyllin ever arise when the drug is prescribed according to my method. On the contrary, it is one of the most satisfactory and reliable of our medicines. The formula given is:

℞. Podophilli....................................gr. ij.

Essentiæ zingiberis..........................ʒ ij.

Spiritus vini recti............................q. s. ad. ℥ ij.

Fiant guttæ.

A teaspoonful to be taken in a wineglassful of water every night at bedtime, or every second, third or fourth night, as required.—DR. HORACE DOBELL, *in British Medical Journal.*

**Action of Quinine, Digitaline and Atropine.**—Dr. Guido Cavazzani has arrived at the following conclusions on this subject: Quinine and atropine have an astringent action upon the peripheral vascular extremities. They correct the vascular dilatation caused by digitalis. Atropine and digitaline are antagonistic—the first giving tone to the terminal vessels and paralyzing the heart, the second producing an opposite effect. These two remedies associated cause considerable slowing of the ventricular contractions of the heart, and much less slowing of the auricular contractions. Quinine and digitaline combined reciprocally increase their force of action. Quinine and atropine neutralize each other as to their action upon the heart. These three remedies given singly may cause a state of collapse, which in quinine is due to ischemia of the heart; in digitaline to its tetanization; and in atropine to its asthenia.—*Medical Press and Circular.*

**No Yellow-Fever Germ.**—Dr. George M. Sternberg, in a recent pamphlet, gives an account of the microscopical investigations of the Yellow-fever Commission of the National Board of Health.

Dr. Sternberg is at present in New Orleans conducting experiments in connection with the other work of the Havana Commission. Though his researches have not been continued long enough to lead to many definite results, he feels justified in announcing that "there is no gross and conspicuous germ or organism either in the blood of yellow-fever patients or in the air of infected localities, which by its peculiar appearance or abundant presence might arrest the attention of the microscopist and cause suspicion that it is the veritable germ of yellow-fever. Thus it seemeth that the peculiar acicular crystals found in the air of infected districts in Havana are a delusion.—*Louisville Medical Herald.*

**Substitute for Tracheotomy.**—Dr. Macewen, of Glasgow, says, in Belleville Medical Journal:

1. Tubes may be passed through the mouth into the trachea not only in chronic but also in acute affections—such as edema glottidis.
2. They can be introduced without placing the patient under an anæsthetic.
3. The respirations can be perfectly carried on through them.
4. Expectoration can be expelled through them.
5. Deglutition can be carried on during the time the tube is in the trachea.
6. Though the patient at first suffers from a painful sensation, yet this passes off, and the parts soon become tolerant of the presence of the tube.
7. The patient can sleep with the tube *in situ*.
8. The tubes, in these cases at least, were harmless.
9. The ultimate results were rapid, complete and satisfactory.
10. Such tubes may be introduced in operations on the face and mouth, in order to prevent blood from gaining access into the trachea, and for the purpose of administering the anæsthetic, and they answer this purpose admirably.

**High Temperature from Constipation.**—A patient in the Massachusetts General Hospital, while convalescing from a mammary abscess, suddenly developed one morning a temperature of 104.5° F. The abscess was rapidly healing, and the temperature during the preceding eight days had not risen above 99° F. The patient, however, had not had a movement from the bowels for four days. An enema of soap-suds was given, and in less than an hour after this had operated, the temperature fell to 100° F., and afterward continued normal. The patient made no complaint, nor was there any phenomenon of any sort to account for the high temperature, unless the constipation would do so. No remedy was used except the enema.—*Boston Medical and Surgical Journal, August* 12, 1880.

**For Nightsweats of Patients suffering with Lung-phthysis.**—Dr. Kuehnborn prescribed with astonishing success a dusting-powder consisting of 3 parts salicylic acid, 10 parts starch and 87 parts Venetian talc, which was dusted all over the body, the skin of the body if too dry, being first rubbed with alcohol and tannin so as to make the powder adhere. In order to prevent the irritation and cough so usually brought about by the dust of the acid it is necessary for the patient to press a cloth on mouth and nose during the dusting. The use of the powder prevented nightsweats in every case without causing any other inconvenience.—*American Journal of Pharmacy.*

**Friction in Insomnia.**—Nervous persons are more than all others subject to sleeplessness. To obtain a little sleep they have recourse to narcotics which always end by having a pernicious influence on the health. We can recommend to such a very simple method, and which infallibly procures the repose many seek by other means: this is the rubbing of the body, or friction for some minutes before retiring, either with a piece of coarse woollen cloth, or, if preferred, with a friction brush.—*La Lancette Belge.*

**Neuralgia of the Testis.**—Prof. Wm. A. Hammond contributes an article to the St. Louis Journal of Medicine, May, 1880, on Neuralgia of the Testis, in which he relates the histories of two cases, which were successfully treated by a method not hitherto employed in this affection. The first patient, æt. 47, had suffered more or less severely for over fifteen months. He admitted that the affection was originally, in all probability, induced by excessive venereal indulgence, but insisted that since the inception of the disease he had been extremely temperate in this direction. There was no evidence of syphilis. The pain was of a sharp, lancinating character, not confined to the testicle, but extending up the cord as high as the external abdominal ring. The cremaster muscle was, during the continuance of the paroxysms, the subject of strong spasm. Walking increased the pain, and sometimes brought on a seizure. The patient had tried all sorts of treatment without relief, and Dr. Hammond had already given an unfavorable prognosis, when the idea struck him that possibly strong pressure applied to the spermatic cord, so as to compress the nerves, might arrest the spasms. He extemporized a compressor out of an ordinary test-tube holder and a strong India rubber band, and applied it while the patient was suffering from a severe paroxysm, so as to compress the cord up as high as possible. So far from adding to the pain he was suffering, the immediate effect was a decided amelioration, but after a few minutes the pain began to increase, and soon became more intense than before. The pressure was then increased by squeezing the blades of the instrument together with the fingers, and the pain stopped at once. The instrument was kept applied for fifteen minutes longer. Six hours afterward there had been no return of the pain, and to the present date the patient has remained entirely free from all pain. Sensation, which was destroyed by the pressure, has returned to the scrotum and testicle, and there are normal erections and sexual desires. The other patient, æt. 38, had suffered from the neuralgia for three months, and at the time he came under observation his suffering was particularly acute. In this case there was no reason for suspecting excessive sexual indulgence or syphilitic infection as the cause; apparently it was due to exposure to cold. As in the other case, the right testicle was the seat of the disease. Walking, sitting or standing aggravated the suffering, and only by lying down on his back did he obtain any marked alleviation. Since the inception of the malady, venereal desires had almost entirely ceased, and erections, such as those caused at night by lying on the back, or by distention of the bladder, added greatly to the suffering. Pressure was applied to the cord by means of an apparatus similar to a lemon-squeezer, but so arranged that the blades could be brought closer together or separated by means of a screw passing through them. To be effectual in relieving the pain of a neuralgic testis, Dr. Hammond believes that the pressure must be strong enough to break up the axis-cylinder of the nerves. If less than this, the pain will be aggravated; doubtless, in time, the nerve is restored to a state of integrity, but how long a period is required for the purpose cannot yet be determined.—*New York Medical Record.*

